# Where are we now and where are we headed?

**DOI:** 10.1002/ski2.254

**Published:** 2023-05-25

**Authors:** Ewan A. Langan

**Affiliations:** ^1^ Department of Dermatology University of Lübeck Lübeck Germany; ^2^ Dermatological Sciences University of Manchester Manchester UK

Sitting in my office in Lübeck on a late Spring Friday afternoon, charting the future editorial direction of *Skin Health and Disease*, the words of the late former German chancellor Helmut Schmidt reverberate in my thoughts. Loosely translated, he once quipped that ‘If you have visions, you need to see a doctor.’

Joking aside, having a vision is crucial to organise and plan our direction of travel. *Skin Health and Disease*, the first new journal from the British Association of Dermatologists in over half a century, started as a vision. The Society recognised the importance and potential of a fully open access Dermatology journal and, under the leadership of George Millington and his editorial team, have delivered an international, multidisciplinary journal covering all aspects of dermatology from basic science to translational and clinical research. The journal has published 10 issues to date, including two special editions centred on psychodermatology^1^ and skin cancer.^2^

As I take over at the helm, is it already mission accomplished?

Well, we've come a long way in the last 3 years but there's still plenty of work ahead. Although the journal is indexed and searchable on PubMed Central, we are applying to be indexed on Medline. We have also applied to be indexed on Web of Science, which will allow us to gain our first impact factor next year. However, we recognise that impact factor is not the only measure of the success of journals. We are accessible worldwide and the publication reporting new onset vitiligo following COVID‐19 infection was downloaded 5281 times. In 2022, we had 77 820 downloads in total.

With new article categories, including Scientific/Research letters and Images in Dermatology and a clear social media presence we're striving to attract even more high‐quality submissions, hallmarked with robust methodology, scientific validity and reliability. In addition, we've developed a competitive pricing plan, with APC discounts available on several article types and waivers and discounts to corresponding authors based in low‐ and lower middle‐income countries.^3^ Through our Publisher Wiley, SHD has access to 100 read and publish agreements that covers APCs for authors automatically.^4^

Aiming to improve our recognition both nationally and internationally, as a key member of the British Association of Dermatologists' journal family, we want to secure our place as the ‘go to’ open access journal for interdisciplinary and multi‐professional dermatology research. In fact, we can now announce our third special issue which will focus on skin biology and ageing, meeting our commitment to focus on skin health, and its maintenance, throughout the life cycle.

The task of turning our vision into reality has really only just begun and I'm looking forward to the journey.

## AUTHOR CONTRIBUTIONS

**Ewan A. Langan**: Writing–original draft (Lead); Writing–review & editing (Lead).

## CONFLICT OF INTEREST STATEMENT

The author declares no conflicts of interest.

## FUNDING INFORMATION

This article received no specific grant from any funding agency in the public, commercial, or not‐for‐profit sectors.

## ETHICS STATEMENT

Not applicable.

## Data Availability

Data sharing is not applicable to this article as no new data were created or analyzed in this study.
